# 571. Safety and Immunogenicity of INO-4800, a COVID-19 DNA Vaccine as a Primary Series and Booster

**DOI:** 10.1093/ofid/ofab466.769

**Published:** 2021-12-04

**Authors:** Pablo Tebas, Joseph Agnes, Mary Giffear, Kimberly A Kraynyak, Elliott Blackwood, Dinah Amante, Emma Reuschel, Neiman Liu, Mansi Purwar, Aaron Christensen-Quick, Viviane M Andrade, Julie Carter, Gabriella Garufi, Malissa Diehl, Albert Sylvester, Matthew P Morrow, Patrick P Pezzoli, Abhijeet J Kulkarni, Faraz I Zaidi, Drew Frase, Kevin Liaw, Ami Patel, Karen R Buttigieg, John E Ervin, Jan Pawlicki, Elisabeth Gillespie, Igor Maricic, Katherine Schultheis, Hedieh Badie, Timothy A Herring, Keiko O Simon, Trevor R F Smith, Stephanie Ramos, Robert Spitz, Jessica Lee, Michael Dallas, Ami Shah Brown, Jacqueline E Shea, J Joseph Kim, David Weiner, Kate Broderick, Trevor McMullan, Jean Boyer, Laurent Humeau, Mammen P Mammen Jr.

**Affiliations:** 1 Perelman School of Medicine, University of Pennsylvania, Philadelphia, Pennsylvania; 2 INOVIO Pharmaceuticals, Plymouth Meeting, Pennsylvania; 3 Wistar Institute, philadelphia, Pennsylvania; 4 Wistar, Philadelphia, Pennsylvania; 5 Wistar Institute Vaccine Center, Philadelphia, Pennsylvania; 6 National Infection Service, Public Health England, Salisbury, United Kingdom; 7 Alliance for Multispecialty Research - KCM, KANSAS CITY, Missouri; 8 Inovio Pharmaceuticals, Inc., Plymouth Meeting, Pennsylvania; 9 ICON GPHS, Hinckley, Ohio

## Abstract

**Background:**

DNA vaccines are safe, tolerable, elicit humoral and cellular responses, allow for repeated dosing over time, are thermostable at room temperature, and are easy to manufacture. We present a compilation of Phase 1 and Phase 2 data of Inovio’s US COVID-19 DNA Vaccine (INO-4800) targeting the full-length Spike antigen of SARS-CoV-2. A South Korean Phase 2 study is ongoing.

**Methods:**

Participants in the open-label Phase 1 trial received 0.5 mg, 1.0 mg or 2.0 mg intradermally (ID) followed by electroporation (EP) at Days 0 and 28. An optional booster dose was administered >6 months post-dose 2. The Phase 2 further compared the 1.0 mg and 2.0 mg doses against placebo in a total of 401 participants randomized at a 3:3:1:1 ratio. ClinicalTrials.gov identifiers: NCT04336410 and NCT04642638

**Results:**

The majority of adverse events (AEs) related to INO-4800 across both trials were mild in severity and did not increase in frequency with age and subsequent doses. In Phase 1, 78% (14/18) and 84% (16/19) of subjects generated neutralizing antibody responses with geometric mean titers (GMTs) of 17.4 (95%CI 8.3, 36.5) and 62.3 (95% CI 36.4, 106.7) in the 1.0 and 2.0 groups, respectively (Figure 1). By week 8, 74% (14/19) and 100% (19/19) subjects generated T cell responses by Th1- associated IFNγ ELISPOT assay . Following a booster dose, neutralizing GMTs rose to 82.2 (95% CI 38.2, 176.9) and 124.7 (95% CI 62.8, 247.7) in the 1.0 mg and 2.0 mg groups, respectively, demonstrating the ability of INO-4800 to boost (Figure 2). In Phase 2, neutralizing antibody responses demonstrated GMTs of 93.6 (95%CI 77.3, 113.4) in the 1.0 mg dose group and 150.6 (95%CI 123.8, 183.1) in the 2.0 mg dose group (Figure 3).

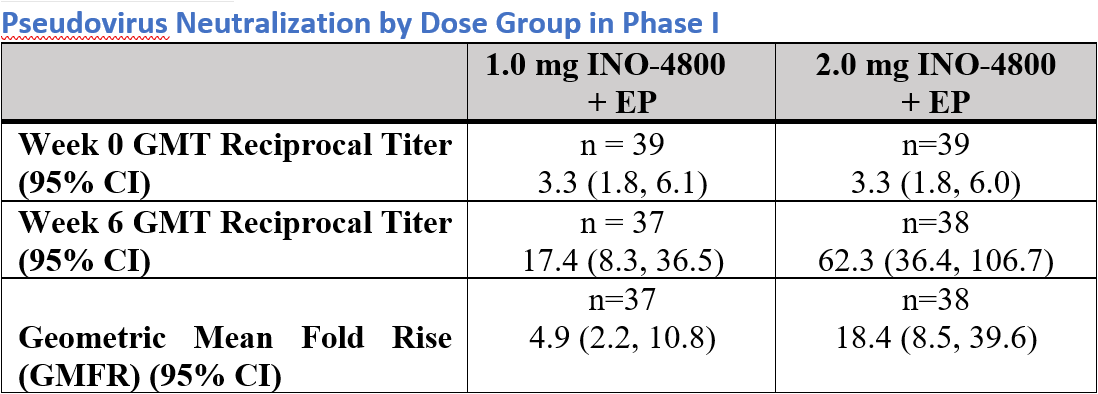

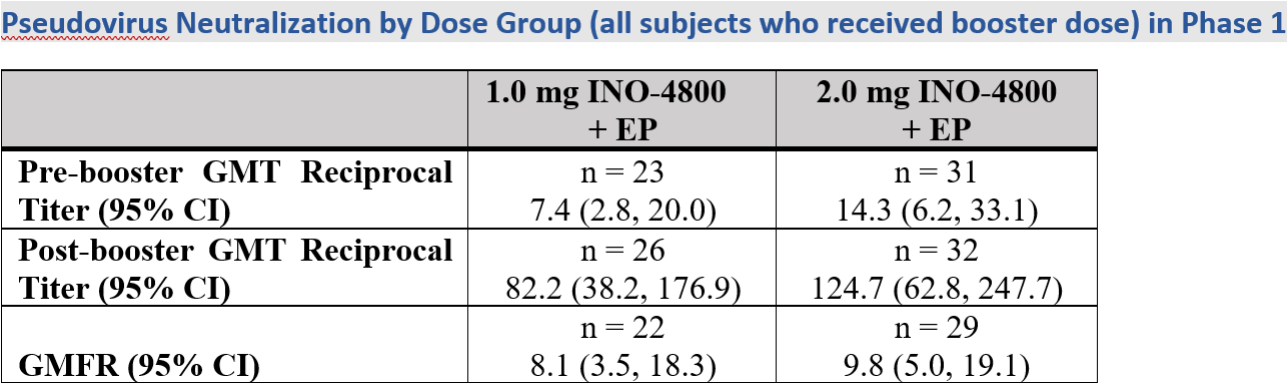

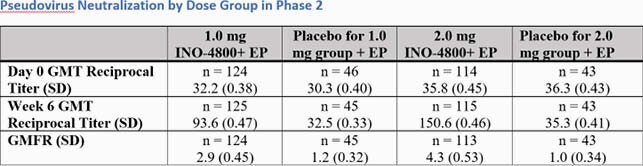

**Conclusion:**

INO-4800 appears safe and tolerable as a primary series and as a booster with the induction of both humoral and cellular immune responses. In addition to eliciting neutralizing antibodies, INO-4800 also induced T cell immune responses as demonstrated by IFNγ ELISpot. Finally, as a homologous booster, INO-4800, when administered 6-10.5 months following the primary series, resulted in an increased immune response without increase in reactogenicity. The 2.0 mg dose was selected for Phase 3 evaluation.

**Disclosures:**

**Joseph Agnes, PhD**, **Inovio** (Employee, Shareholder) **Mary Giffear, BS**, **Inovio Pharmaceuticals, Inc.** (Employee) **Kimberly A. Kraynyak, PhD**, **Inovio Pharmaceuticals** (Employee, Other Financial or Material Support, Stock options) **Dinah Amante, BS**, **Inovio** (Employee) **Emma Reuschel, PhD**, **Inovio Pharmaceuticals** (Employee) **Aaron Christensen-Quick, PhD**, **Inovio Pharmaceuticals, Inc** (Employee) **Viviane M. Andrade, PhD**, **Inovio Pharmaceuticals Inc.** (Employee) **Gabriella Garufi, PhD**, **Inovio Pharmaceuticals, Inc.** (Employee) **Albert Sylvester, MS**, **Inovio** (Employee, Shareholder) **Matthew P. Morrow, PhD**, **Inovio Pharmaceuticals** (Employee) **Patrick P. Pezzoli, BS**, **Inovio Pharmaceuticals, Inc.** (Employee) **Jan Pawlicki, PhD**, **Inovio Pharmaceuticals** (Employee) **Elisabeth Gillespie, PhD**, **Inovio Pharmaceuticals, Inc.** (Employee) **Katherine Schultheis, MSc**, **Inovio Pharmaceuticals** (Employee) **Hedieh Badie, PhD**, **INOVIO Pharmaceuticals** (Employee) **Timothy A. Herring, MPH**, **Inovio Pharmaceuticals, Inc.** (Employee, Other Financial or Material Support, Own stock in the company) **Keiko O. Simon, PhD**, **Inovio Pharmaceuticals** (Employee) **Trevor R. F. Smith, PhD**, **Inovio** (Employee, Shareholder) **Stephanie Ramos, PhD**, **Inovio Pharmaceuticals** (Employee) **Jessica Lee, MPH**, **Inovio Pharmaceuticals** (Employee) **Michael Dallas, PhD**, **Inovio Pharmaceuticals, Inc.** (Employee, Shareholder) **Ami Shah Brown, PhD**, **Abbot Laboratories** (Shareholder)**IBB Biotech ETF** (Shareholder)**Inovio Pharmaceuticals** (Employee)**J & J** (Shareholder)**Moderna** (Shareholder) **Jacqueline E. Shea, PhD**, **Inovio Pharmaceuticals** (Employee, Shareholder) **J Joseph Kim, PhD**, **Inovio** (Employee) **David Weiner, PhD**, **Inovio** (Board Member, Grant/Research Support, Shareholder, I serve on the SAB in addition to the above activities) **Kate Broderick, PhD**, **Inovio** (Employee) **Trevor McMullan, MSc**, **Inovio** (Shareholder) **Jean Boyer, PhD**, **Inovio** (Employee) **Laurent Humeau, PhD**, **Inovio Pharmaceuticals** (Employee) **Mammen P. Mammen Jr., MD**, **Inovio Pharmaceuticals** (Employee)

